# Marble Bone Disease: A Rare Bone Disorder

**DOI:** 10.7759/cureus.339

**Published:** 2015-10-02

**Authors:** Eswaran Arumugam, Maheswari Harinathbabu, Ranjani Thillaigovindan, Geetha Prabhu

**Affiliations:** 1 Prosthodontics, Thai Moogambigai Dental College and Hospital; 2 Oral Medicine and Radiology, Siva Multi Speciality Dental Clinic

**Keywords:** osteopetrosis, marble bone disease, autosomal recessive, dense sclerotic bone

## Abstract

Osteopetrosis, or marble bone disease, is a rare skeletal disorder due to a defective function of the osteoclasts. This defect renders bones more susceptible to osteomyelitis due to decreased vascularity. This disorder is inherited as autosomal dominant and autosomal recessive. Healthcare professionals should urge these patients to maintain their oral health as well as general health, as this condition makes these patients more susceptible to frequent infections and fractures. This case report emphasizes the signs and symptoms of marble bone disease and presents clinical and radiographic findings.

## Introduction

Osteopetrosis (literally "stone bone," also known as marble bone disease or Albers-Schonberg disease) is an extremely rare inherited disorder where the bones harden and become denser. The disorder can cause osteosclerosis. The estimated prevalence of osteopetrosis is 1 in 100,000 to 500,000. It presents in two major clinical forms—a benign autosomal dominant form and a malignant autosomal recessive form. The autosomal dominant adult (benign) form is associated with few, if any, symptoms, and the autosomal recessive infantile (malignant) form is typically fatal during infancy or early childhood if untreated [1].

A rarer autosomal recessive (intermediate) form presents during childhood with some signs and symptoms of malignant osteopetrosis. In especially rare cases, osteopetrosis may present as lethal, transient infantile, and post-infectious forms. Most children born with the malignant form of osteopetrosis die during infancy. Due to better medical care, the life expectancy of these patients has increased in recent years [2-3].

Oral problems associated with osteopetrosis are delayed tooth eruption, absence of some teeth, malformed teeth, enamel hypoplasia, disturbed dentinogenesis, hypomineralisation of enamel and dentin, a propensity for tooth decay, defects of the periodontal membrane, thickened lamina dura, mandibular protrusions, and the presence of odontomas. Tooth removal should be limited as patients increase in age, due to the risk of bone fractures and osteomyelitis [2]. However, dental development and tooth eruption can become a practical and medical problem [3]. To improve oral hygiene in these patients, (especially in areas of exposed mandibular bone), a 0.2% chlorhexidine formulation can be used. Fluoride applications can be done to decrease the susceptibility to dental caries [4].

## Case presentation

A 23-year-old female came to the department of prosthodontics complaining of missing teeth and wanted to have her missing teeth replaced. Her history revealed missing teeth since age five, leading to an inability to eat and speak properly from that time forward. The patient had no relevant history of hypertension, asthma, tuberculosis, drug allergy, bleeding disorders, or cardiovascular disease. The patient has a history of anemia and has been on an iron supplement regimen since age five.

The patient also has a history of oral infection and associated illnesses. The patient experienced a traumatic extraction of her right lower molar five years prior in the right lower back jaw due to caries, which was followed by a severe infection in that area in which the wound did not heal for one month. She was treated in the hospital for 15 days during which time she underwent surgical debridement of the wound and received intravenous antibiotics. The patient has no other relevant surgical history. The patient’s parents and only sister are apparently healthy. On clinical examination, the patient was calm, cooperative, and well-oriented with a normal build and was moderately nourished. The patients presented with a pallor of the conjunctiva and nails. There were no detectable systemic diseases present.

Patient consent was obtained to use her medical history, photographs and radiographs for the study purpose. 

**Figure 1:**
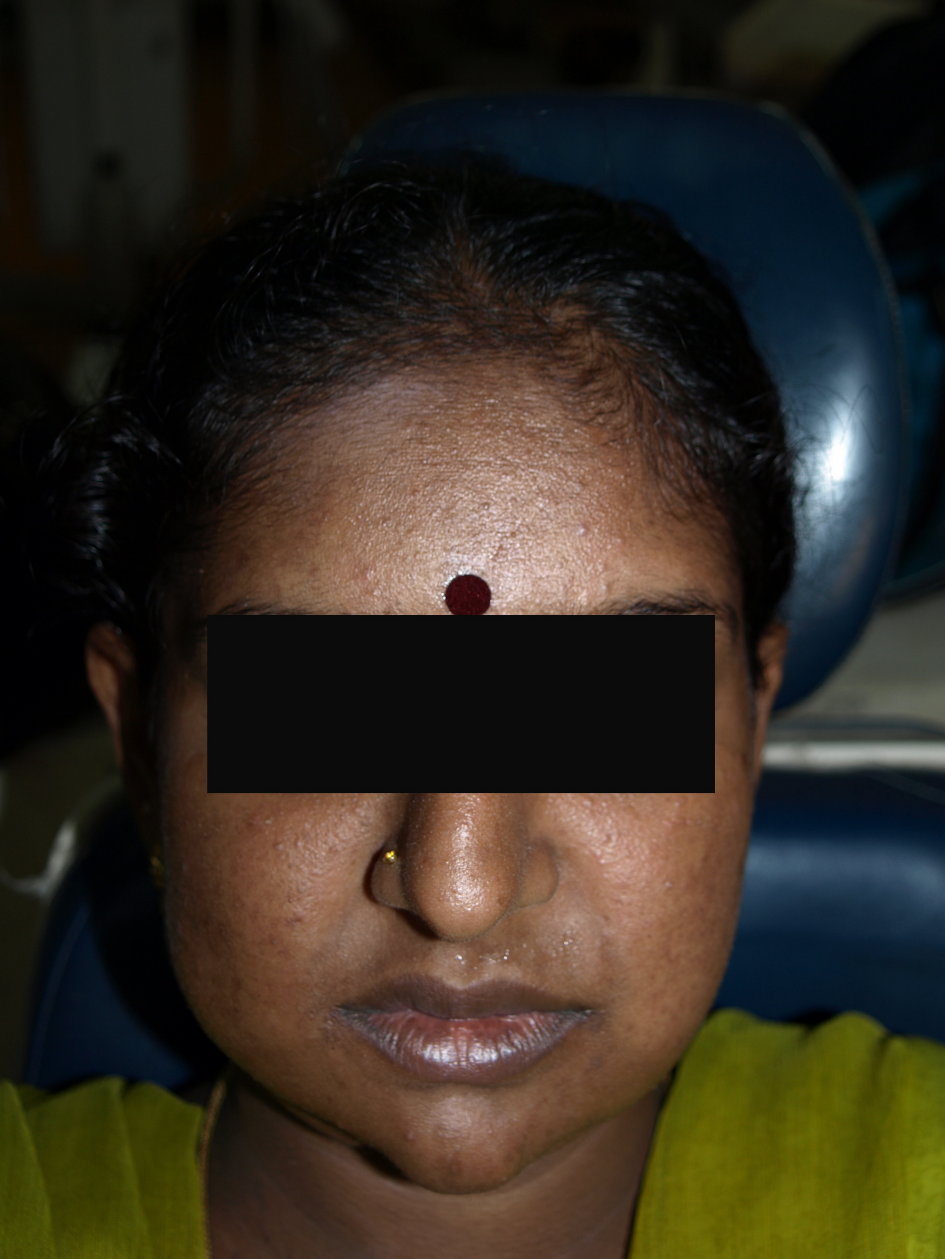
Facial asymmetry

Extraorally, there was facial asymmetry in the right side of the face, as seen in Figure 1.

**Figure 2:**
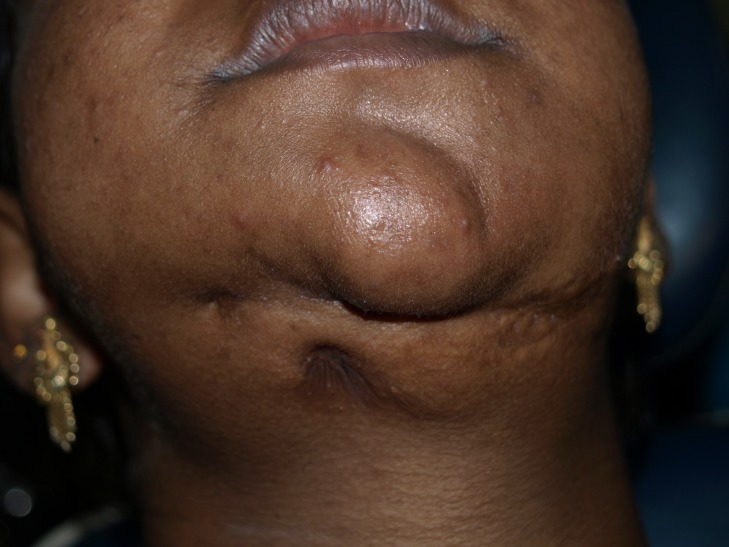
Scar in the lower border of mandible

Patient has a scar in the lower border of the mandible, as seen in Figure 2.

**Figure 3:**
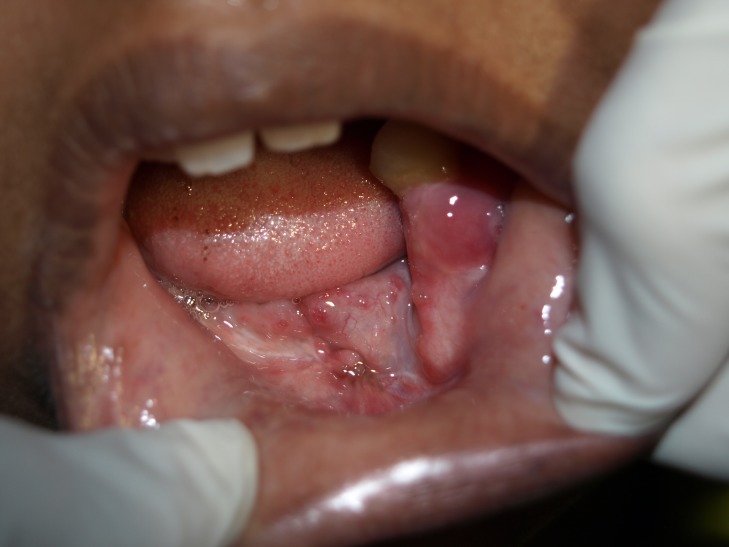
Intraoral missing tooth

One right submandibular lymph node is palpable, tender, firm in consistency, freely mobile, and measuring around 1.5 cm × 1.5 cm in size. The patient’s ability to open her mouth is restricted, with an interincisal distance of 35 mm. Limited jaw movements were noted. Teeth missing were 15, 16, 27, 31, 32, 33, 34, 35, 41, 42, 43, 44, 45, 46, and 47 (Figure 3).

**Figure 4:**
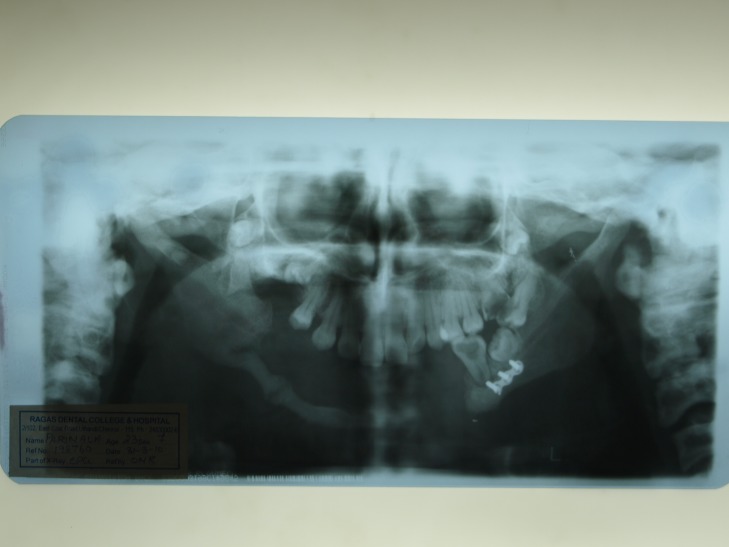
OPG shows fracture and resection

Urine and blood routine investigations revealed normal results. Orthopantomogram reveals the presence of surgical plating in relation to the 36 and 37 periapical regions, and a 4 cm discontinuity in the lower cortical region of the mandible in the left premolar region. The patient has a resected mandible leaving a 1 cm cortical border in the right side of the mandible, and a thickening of the fractured edges in the right body of the mandible with an interposed 0.5 cm radiolucency between the fractured edges, as noted in Figure 4.

**Figure 5:**
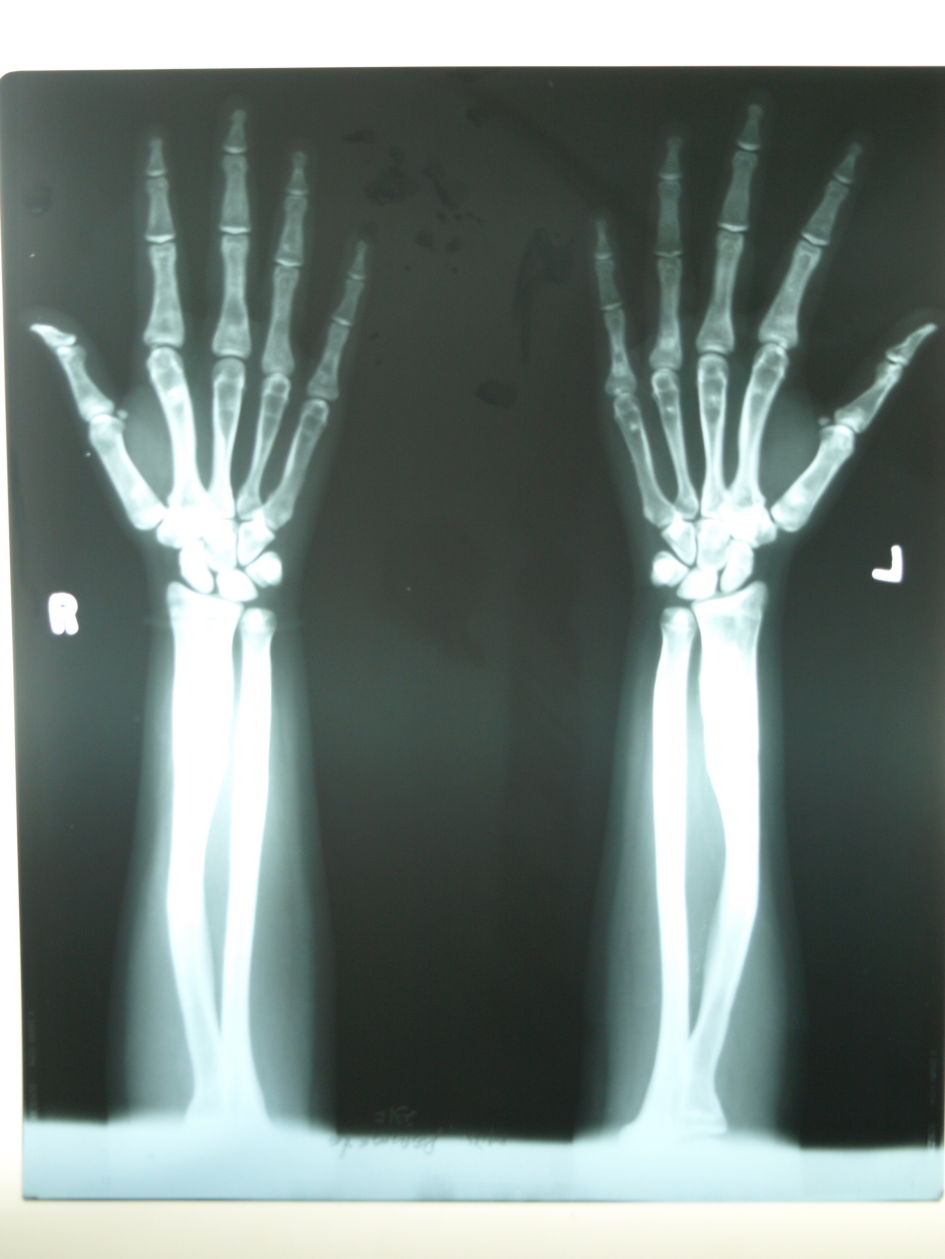
Radiograph of hands showing increased radio-opacity

**Figure 6:**
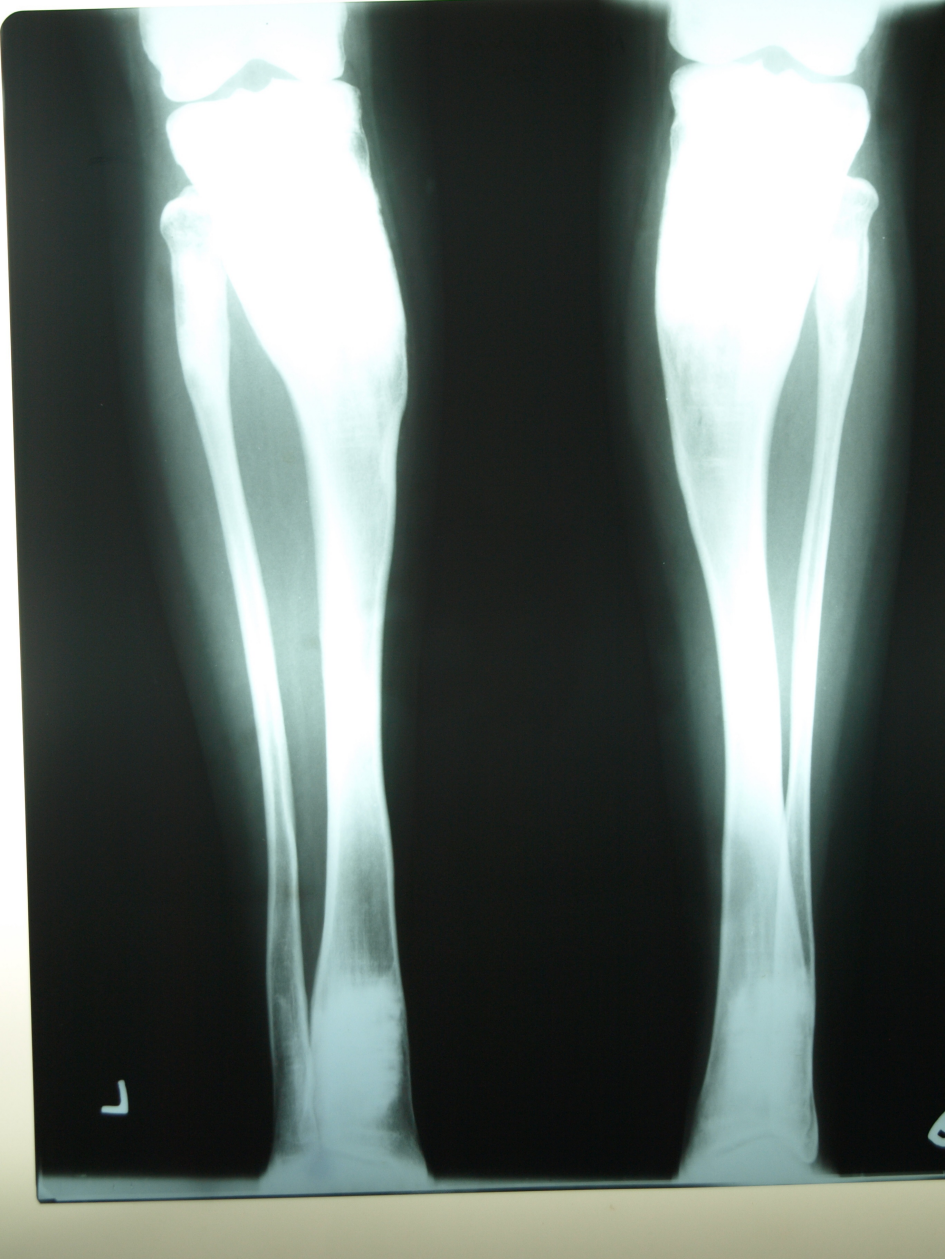
Radiograph of legs showing increased radio opacity

A radiograph of the patient’s hands (Figure 5) and legs (Figure 6) reveals the obliteration of the normal marrow spaces with increased radio-opacity of the bone, depicting a “Candle stick” appearance.

**Figure 7:**
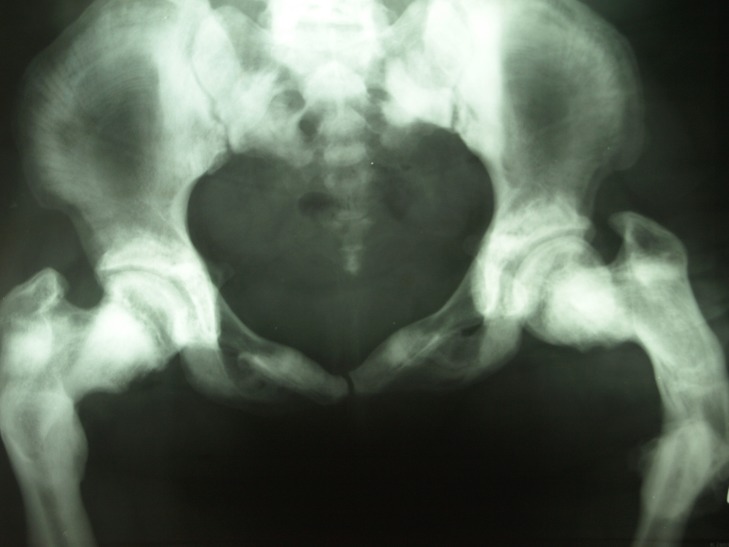
Radiograph of pelvic bones showing fracture

Figure 7, a radiograph of the pelvic bone, also reveals an increased radio-opacity of the bone, with a healing fracture site in the left femur, which also denotes the site of malunion due to improper positioning of the fracture edges.

A histopathological examination of the buccal cortical specimen from the extracted 37 region reveals dense sclerotic bone with most of the marrow spaces replaced with bone. The bone is avascular with some islands of cartilage. Creatine kinase, a biochemical marker of osteopetrosis, was present and confirmed the diagnosis of marble bone disease. 

## Discussion

Normal bone growth is achieved by a balance between bone formation by osteoblasts and bone resorption (the breakdown of the bone matrix) by osteoclasts. In osteopetrosis, the number of osteoclasts may be reduced, normal, or increased. More importantly, osteoclast dysfunction mediates the pathogenesis of this disease. The exact mechanism is unknown. However, a deficiency of carbonic anhydrase in the osteoclasts is noted. The absence of this enzyme causes inhibited hydrogen ion pumping in osteoclast cells and this, in turn, causes defective bone resorption because an acidic environment is needed for the dissociation of calcium hydroxyapatite from the bone matrix. Hence, bone resorption fails while bone formation persists, causing an imbalance and excess of bone [1].

Despite this excess bone formation, people with osteopetrosis tend to have bones that are more brittle than normal. Mild osteopetrosis may cause no symptoms, and present no problems. However, serious forms of osteopetrosis can result in stunted growth, deformity, and an increased likelihood of fractures. Patients can also suffer anemia, recurrent infections, hepatosplenomegaly, and extramedullary hematopoiesis (due to bone expansion leading to bone marrow narrowing). Osteopetrosis can also result in blindness, facial paralysis, and deafness, due to the increased pressure put on the nerves from the excess bone formation encroaching on the cranial nerve foramina [2].

As osteopetrosis patients age, dental development and tooth eruption become a practical and medical problem [3]. To improve oral hygiene in these patients (especially in areas of exposed mandibular bone), a 0.2% chlorhexidine formulation can be used. Fluoride applications can be done to decrease the susceptibility to dental caries [4].

Dental abnormalities may be attributed to the pathological changes in osteopetrosis. Patients with the disease seem to be especially susceptible to caries. The possible contributing factors to bone necrosis and dental caries are the constriction of the canals housing neurovascular bundles that supply the teeth and jaws, along with the obliteration of the marrow cavities and the dental pulp chambers. Other dental changes may include the delayed eruption and early loss of teeth, enamel hypoplasia, malformed roots and crowns, and a thickening of the lamina dura [5].

The avascularity of the marrow spaces and the partial obliteration of the mandibular canal appear to be factors contributing to a reduced resistance to infection of the bone tissue. These patients are susceptible to infection as a result of granulocytopenia. Severe infections have the potential to run a protracted course due to the accompanying severe anemia and neutropenia [6].

Most patients fail to grow, and death can occur at an early age as a result of severe chronic anemia, bleeding, and/or infection [7]. Abnormal bone formation and fibrous tissue replace the bone marrow space, leading to decreased hematopoiesis. Extramedullary hematopoiesis can occur, resulting in leukoerythroblastic anemia and thrombocytopenia. The patient’s liver and spleen will enlarge. Hemolysis resulting from hypersplenism will worsen the anemia and thrombocytopenia [8]. Genetic consultation is important, and the prenatal diagnosis of osteopetrosis early in pregnancy is an indication for termination of the pregnancy [9-10].

### Differential diagnoses

The differential diagnoses include other disorders which can cause diffuse osteosclerosis, such as hypervitaminosis D, hypoparathyroidism, Paget's disease, diffuse bone metastasis of breast or prostate cancer (which tend to be osteoblastic while most metastases are osteolytic), intoxication with fluoride, lead, or beryllium, and hematological disorders, such as myelofibrosis, sickle cell disease, and leukemia [11-12].

### Treatment

The only durable cure for the form of osteopetrosis that affects the osteoclasts (which is more common) is a bone marrow transplant [3]. If complications occur in children, patients can be treated with vitamin D. Interferon-gamma has also been shown to be effective, and it can be associated with vitamin D. Erythropoietin has been used to treat the associated anemia.

Corticosteroids may alleviate both the anemia and stimulate bone resorption. Fractures and osteomyelitis can be treated as usual. Both the adult and childhood forms of osteopetrosis may benefit from Actimmune® (interferon gamma-1b) injections. Actimmune delays the progression of malignant infantile osteopetrosis because it causes an increase in bone resorption and in red blood cell production. In the present patient case, oral prophylaxis was performed and oral hygiene measures instituted. A loss of denture support due to resected bone in the left side of the mandible was noted [11]. The department of prosthodontics was consulted regarding the replacement of the missing teeth with a mandibular overlay denture. The treatment of osteopetrosis should be as conservative as possible. The patient was referred to a general hospital regarding bone marrow transplantation as a treatment option.

## Conclusions

Prevention of osteopetrosis is not possible as it is a genetically mediated disorder. Proper prenatal screening for the genetic disorder followed by maintenance and supportive therapy can help the patient lead a near normal life. The ultimate goal of the treatment should not only be focusing oral health but also the physical rehabilitation of the patient.
